# Desmoplastic small round cell tumour in a 74 year old man: an uncommon cause of ascites (case report)

**DOI:** 10.1186/1746-1596-6-55

**Published:** 2011-06-23

**Authors:** Andrew J Heikkila, Ally PH Prebtani

**Affiliations:** 1 McMaster University, Department of Anesthesia, Health Sciences Centre 2U1, 1200 Main Street West, Hamilton, Ontario, Canada; 2 Hamilton General Hospital, 237 Barton Street, Hamilton, Ontario, Canada

## Abstract

A rare case is provided of a 74 year old man who presented with ascites of unknown etiology. CT scan of the abdomen revealed extensive omental caking, and omental biopsy cytogenetics showed findings in keeping with a diagnosis of desmoplastic small round cell tumour (DSRCT). This case is unique in that it involves a significantly older patient, negative WT1 immunohistochemical staining, and negative cytology. Despite repeated paracenteses and fluid management, the patient died in hospital secondary to renal complications.

## Background

DSRCT is an exceedingly rare malignancy, first described by Gerald and Rosai in 1989. Characterized by a predilection for young males, it typically presents as an intra-abdominal mass with multiple intra-peritoneal implants, and has a dismal 5-year survival rate. Recent years have brought forth invaluable information regarding the immunohistochemical and cytogenetic features of this tumour. Below we present a recent case of DSRCT in a 74 year old man who presented with ascites. Investigations interestingly revealed negative WT1 staining and cytology. This is followed by a discussion of key clinical and investigational findings that aided in diagnosis, as well as an approach to unusual causes of ascites.

## Case Presentation

A 74 year old man presented to hospital with a six week history of increasing abdominal girth, worsening umbilical pain, decreased appetite, and a six month history of significant unintentional weight loss. His past history was significant for an excised rectal tubular adenoma, Benign Prostatic Hypertrophy, and Gastroesophageal Reflux Disease. He was a non-smoker and a non-drinker. He had recently been prescribed Spironolactone 25 mg daily for his ascites and he took Rabeprazole 20 mg daily. Initially, the patient's blood pressure was 124/80, pulse 115 and regular, respiratory rate 20, and he was afebrile. The abdomen was distended with bulging flanks. There was a fluid wave and shifting dullness. There were no signs of chronic liver disease. Cardiovascular exam was normal, with no murmurs or extra heart sounds, and the JVP was 2 cm. Breath sounds were heard to the lung bases bilaterally with no crackles. There was no peripheral edema and no evidence of lymphadenopathy of the head and neck or groin.

**Figure 1:**
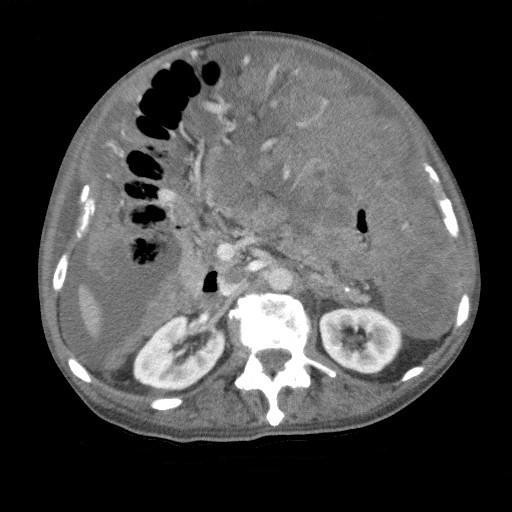
**Axial 5 mm CT slice, at the level of the renal hilum, demonstrating dense omental caking, high density fluid with increased omental vascularity**.

**Figure 2:**
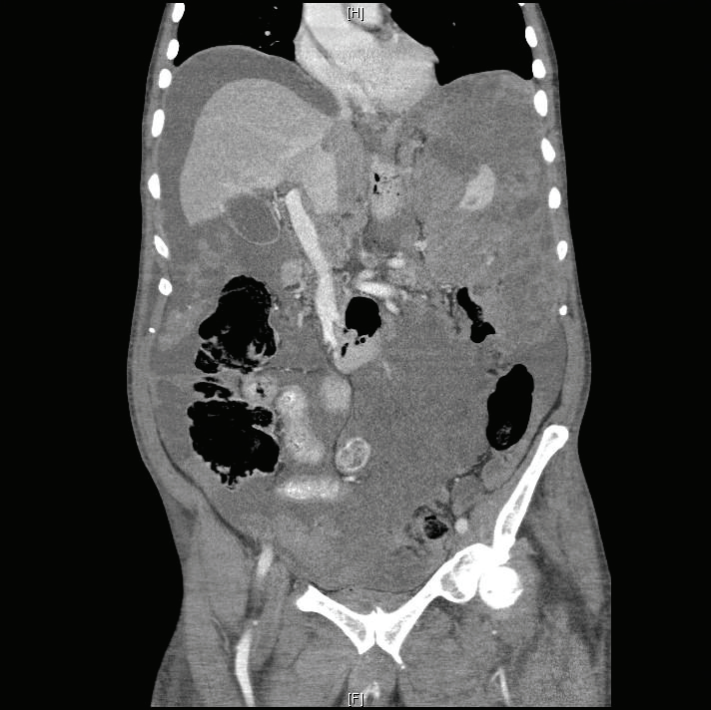
**Coronal CT view demonstrating marked omental caking with interspersed high density fluid filling the left hypocondrium and left peritoneal region**.

A computed tomography (CT) scan of the abdomen showed extensive omental caking and ascites (Figures 1 and 2). This was seen as heterogenous nodular enhancement of the omentum with marked dense free fluid in the abdominal and pelvic cavities. The liver and bowels were unremarkable. No bony lesions were seen. Multiple enlarged paraaortic lymph nodes with the absence of central hilar fat were noted. CT of the chest did not demonstrate any lesions.

Therapeutic and diagnostic paracenteses were performed, which revealed visibly bloody peritoneal fluid. Analysis was negative for malignant cells on both occasions. The differential cell count was: monocytes 14%, lymphocytes 67%, and neutrophils 19%. The serum and fluid albumin values were 33 g/L and 25 g/L, respectively, giving a serum-ascites albumin gradient (SAAG) of 8 g/L, suggestive of non-portal hypertension ascites. Cultures of the fluid were negative for infection.

**Figure 3:**
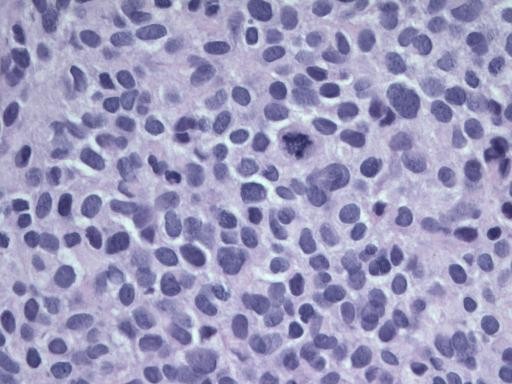
**Microphotograph representative of the core biopsy material demonstrating a diffuse population of small undifferentiated malignant cells**. [Hematoxylin and Eosin stain, 400 ×.]

Ultrasound guided omental biopsies were performed. Microscopic analysis showed neoplastic small round blue cells arranged in sheets, without a desmoplastic stroma (Figure 3). The cells possessed large hyperchromatic nuclei and amphophilic cytoplasm. There were scattered mitoses, less than 1 per 10 HPF with foci of microscopic necrosis.

**Figure 4:**
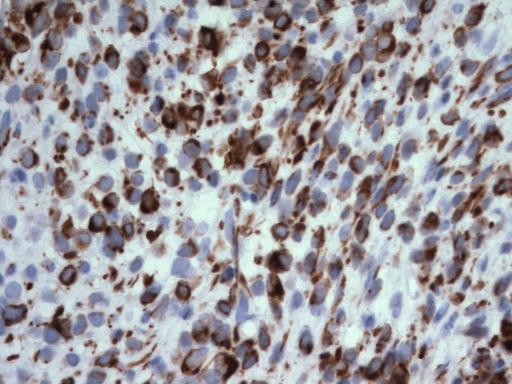
**Microphotograph demonstrating cytoplasmic diffuse and punctate staining of malignant cells for desmin**. [400 ×.]

**Figure 5:**
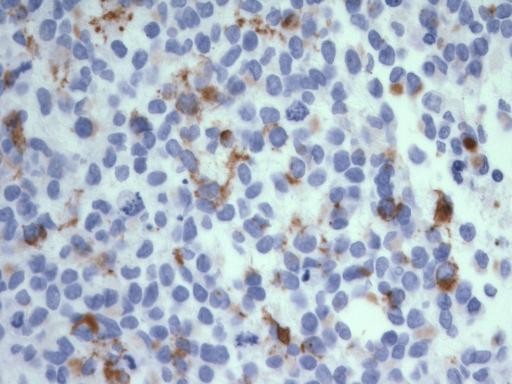
**Microphotograph demonstrating cytoplasmic, punctate staining of malignant cells for Cam 5.2**. [400 ×.]

**Figure 6:**
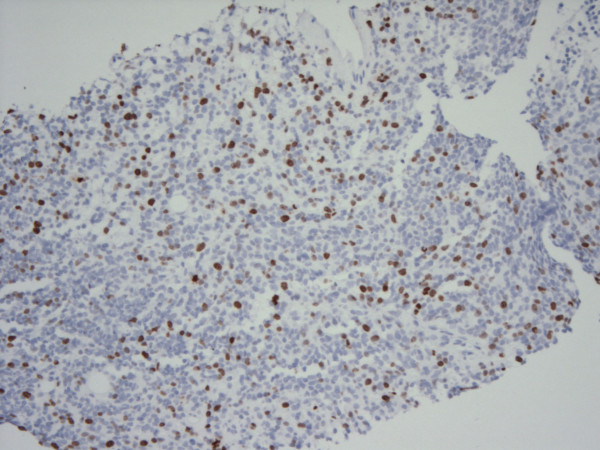
**Microphotograph demonstrating positive Ki-67 staining in nearly 35% of tumour cells**. [MIB-1 stain, 100 ×.]

Immunohistochemistry showed a strong positive desmin staining of cytoplasm and punctate (Figure 4). There were scattered cells positive for CAM 5.2, punctate pattern (Figure 5), as well as rare EMA positive cells. Staining for Ki-67 with MIB-1 was positive in up to 35% of tumour cells (Figure 6), and the cells were negative for WT1.

Several days later, cytogenetics results were available and demonstrated the presence of EWS/WT-1 fusion transcriptase arising from the (11;22) translocation, characteristic of DSRCT.

By the third week of admission, despite repeated therapeutic paracenteses, the patient had significantly deteriorated. He became bed bound, anorexic, and weak. He developed impressive peripheral edema with ongoing tense ascites, leading to intravascular fluid depletion. He was hypotensive and in acute renal failure with hyperkalemia. The decision was made for comfort measures only after the final pathological diagnosis. He passed away shortly thereafter. An autopsy was not performed.

## Discussion

DSRCT is an exceptionally rare malignancy, first described by Gerald and Rosai in 1989 [1]. Characterized by a predilection for young males, it typically presents as an intra-abdominal mass with multiple intra-peritoneal implants, and has a 5-year survival rate of 15% [2]. Our patient died approximately 7 months following the onset of symptoms.

As with all medical diagnoses, that of DSRCT involves a thorough history, physical examination and series of investigations. Our patient certainly did not fit the usual demographics for DSRCT, a disease that typically affects adolescents and young adults. However, his clinical findings of abdominal pain, anorexia, and ascites, though non-specific for DSRCT, represent three of the more commonly described signs and symptoms of this malignancy [3].

In Cao et al. report of 18 DSRCT cases, five of the patients presented with ascites [3]. Lae et al. documented ascites occurring in only two of 18 DSRCT cases [4]. Thus, ascites is a clinical component of this disease, though it rarely occurs in isolation. Furthermore, ascites has a broad differential diagnosis which warrants a methodical workup.

Cirrhosis accounts for more than 85% of cases of ascites, with the remaining 15% caused by congestive heart failure, malignancy, and other less common diseases. Malignancy accounts for approximately two-thirds of non-cirrhotic ascites [5]. When confronted with ascites, a thorough clinical history is essential in differentiating hepatic from non-hepatic causes. A discussion of risk factors for liver disease, including alcohol use and viral hepatitis, as well as a history of cardiac disease should be sought. An episode of new onset ascites accompanied by abdominal pain is suspicious for malignancy [6].

Physical examination is equally important in the workup of ascites. Fluid wave and shifting dullness are among the most specific markers of ascites (90% and 72%, respectively), whereas flank dullness, bulging flanks, and shifting dullness are the most sensitive (84%, 81%, and 77%, respectively) [7]. Examination beyond the abdomen may demonstrate palmar erythema, gynecomastia, and spider nevi, suggestive of liver disease. An elevated jugular venous pressure suggests congestive heart failure or constrictive pericarditis. Generalized edema, particularly of the face and upper limbs, suggests nephrotic syndrome. The absence of these unique clinical findings pushes more towards a malignant etiology.

Malignant ascites often occurs when the malignancy is advanced or recurrent. Urinary bladder and ovarian malignancies, as well as cancers of the colon, stomach and pancreas can cause peritoneal carcinomatosis with associated ascites [8]. DSRCT is an exceedingly rare cause of malignant ascites. However, features of ascitic fluid can narrow the differential within this malignancy-related group.

Ascitic fluid analysis should be routinely performed in any patient with new-onset ascites. 20 mL of fluid should be placed in two blood culture bottles at the bedside, and another 10 mL should be sent for cell count and albumin. Fluid cytology may be ordered if malignancy is high on the differential. A serum albumin should be drawn at the same time.

The gross appearance of ascitic fluid can be modestly helpful in elucidating the etiology. 20% of malignant ascites fluid is grossly bloody, as in our case, though more than half of such cases are due to hepatocellular carcinoma [8]. Only 5% of cirrhotic ascites is bloody. Cell count and differential are useful in identifying spontaneous bacterial peritonitis, although the white cell count is generally also elevated in malignancy-related ascites [6]. Cytology may be extremely valuable in suspected malignancy, being positive in 97-100% of cases of peritoneal carcinomatosis, but was surprisingly negative in our case [6].

The SAAG, a parameter of oncotic pressure gradient, has been used for more than two decades to distinguish portal hypertension-related ascites from all other causes with a diagnostic accuracy of 97% [5]. An elevated SAAG (11 g/L or greater) correlates with portal hypertension, whereas a low gradient indicates no portal hypertension. Table 1 outlines common pathologies associated with both high and low SAAG values [6]. In our case, a SAAG of 8 g/L, when combined with the patient's clinical features, certainly favoured a malignant diagnosis.

**Table 1:** Ascites Etiologies by Serum-Ascites Albumin Gradient (SAAG)

Ascites Etiologies by SAAG	
**SAAG ≥ 11 g/L**	**SAAG < 11 g/L**
Cirrhosis	Peritoneal carcinomatosis
Congestive heart failure	Pancreatitis
Alcoholic hepatitis	Nephrotic Syndrome
Hepatic metastases	Serositis
Portal vein thrombosis	Tuberculosis
Budd-Chiari syndrome	

An elevated SAAG (11 g/L or greater) correlates with portal hypertension, whereas a low gradient indicates no portal hypertension [6].

Tumour analysis, most notably immunohistochemical and cytogenetic studies, has enhanced our ability to diagnose DSRCT and has identified it as a unique clinical entity. Microscopically, the biopsy samples in our case fit the well-documented description of small round blue tumour cells, although there was minimal surrounding desmoplastic stroma. Typically, the tumour cells are separated by plentiful stroma [9].

Histologically and cytogenetically, DSRCT shares features with other round cell tumours including small cell carcinoma, mesothelioma, Wilms' tumour, and Ewing's sarcoma/peripheral neuroectodermal tumours (PNET). DSRCT cells are normally reactive for keratin, epithelial membrane antigen, desmin, vimentin, and neuron-specific enolase [4,10-15]. Small cell carcinoma often lacks desmoplastic stroma and is not immunoreactive with desmin [16]. In our case, desmin was positive and demonstrated the unique punctate and cystoplasmic staining. Interestingly, WT1 was negative, a finding unusual for DSRCT. More than 70% of DSRCT cases are positive for WT1 [17], a finding which has also been shown with mesothelioma and Wilms' tumour [18]. Mesothelioma can often be distinguished by positive calretinin expression [17,19,20], while Wilms' tumour lacks the characteristic chromosomal translocation and occurs in a much younger patient population [9]. In our patient, cytogenetic studies revealed the typical t(11;22) translocation involving the short arm of chromosome 11. Ewing's sarcoma, which shares many features with DSRCT, is distinguished by a similar translocation which instead involves the long arm of chromosome 11 [21].

## Conclusions

In summary, DSRCT is a rare and highly aggressive, cytogenetically unique malignancy. It represents an exceedingly uncommon cause of ascites. The above case shared many features with those previously reported in the literature, with the exceptions of significantly older patient age, negative WT1 immunohistochemical staining, and negative cytology. A methodical diagnostic strategy, moving from history to imaging, ascitic fluid studies and tumor analysis, can reliably make the diagnosis of DSRCT.

## Consent

Written informed consent was obtained from the patient's family for publication of this case report and any accompanying images. A copy of the written consent is available for review by the Editor-in-Chief of this journal.

## Competing interests

The authors declare that they have no competing interests.

## Authors' contributions

AH participated in collecting clinical data and images and drafting the manuscript. AP participated in its design and coordination and helped to draft the manuscript. All authors have read and approved the final manuscript.
